# Design and methodology of the Geo-social Analysis of Physicians’ settlement (GAP-Study) in Germany

**DOI:** 10.1186/s12995-016-0104-y

**Published:** 2016-03-31

**Authors:** David A. Groneberg, Michael Boll, Jan Bauer

**Affiliations:** Institute of Occupational, Social and Environmental Medicine, Goethe University, Theodor Stern Kai 7, 60590 Frankfurt/Main, Germany

**Keywords:** Health care system, Physician, Geospatial analyses, Socioeconomic status

## Abstract

**Background:**

Unequally distributed disease burdens within populations are well-known and occur worldwide. They are depending on residents’ social status and/or ethnic background. Country-specific health care systems - especially the coverage and distribution of health care providers - are both a potential cause as well as an important solution for health inequalities.

**Methods:**

Registers are built of all accredited physicians and psychotherapists within the outpatient care system in German metropolises by utilizing the database of the Associations of Statutory Health Insurance Physicians. The physicians’ practice neighborhood will be analyzed under socioeconomic and demographic perspectives. Therefore, official city districts’ statistics will be assigned to the physicians and psychotherapists according to their practice location. Averages of neighborhood indicators will be calculated for each specialty. Moreover, advanced studies will inspect differences by physicians’ gender or practice type. Geo-spatial analyses of the intra-city practices distribution will complete the settlement characteristics of physicians and psychotherapists within the outpatient care system in German metropolises.

**Results:**

The project “Geo-social Analysis of Physicians’ settlement” (GAP) is designed to elucidate gaps of physician coverage within the outpatient care system, dependent on neighborhood residents’ social status or ethnics in German metropolises.

**Conclusion:**

The methodology of the GAP-Study enables the standardized investigation of physicians’ settlement behavior in German metropolises and their inter-city comparisons. The identification of potential gaps within the physicians’ coverage should facilitate the delineation of approaches for solving health care inequality problems.

## Background

Disparities in the spatial and social accessibility of health care providers have been investigated in several countries over the last decades [1]. Here, most research deals with the spatial accessibility focusing on rural physicians’ shortage, which is present in multiple countries worldwide [2]. Less is known about the distribution of physicians in urban areas, concerning their inner-city spatial distribution and/or their settlement behavior regarding practices’ neighborhood socioeconomic and demographic status [3]. An imbalanced physicians’ settlement could lead to disparities in social accessibility despite spatial accessibility being given [4].

Health care disparities seem to play a critical role in the development of imbalances of population’s disease incidences [5]. Research on this topic is referred to as social epidemiology: Disparities in disease burden or behavioral risk factors between subgroups of population are observed. They differ according to their socioeconomic status (SES) and ethnics/races [6–10]. Such health inequalities constitute a worldwide challenge and investigations for a better understanding of sources and for finding approaches to reduce them have been conducted [6].

Health inequalities exist between countries and between social classes or ethnic groups within the same country. Particularly in developed countries, national reports on population’s health inequalities are published on a regular basis generating general overviews on existing disparities [11, 12]. Mortality rate, childhood mortality and life expectancy are inversely correlated with the populations’ SES in most of the developed countries [13, 14]. Compelling evidence for analogous correlations have been found for coronary heart diseases [15, 16], diabetes mellitus type 2 [17] and different types of cancer [18, 19]. On the level of health risks factors, smoking behavior and alcohol abuse were predominantly identified to be significantly SES dependent [20].

Fewer studies have focused on distribution disparities of health care providers. However, the main focus has been put on existing or upcoming rural physician’s shortages, which occur in most of the developed countries [21–26]. Therefore, little is known about the distribution of physicians and other health care providers regarding the socioeconomics and demographics of their practices’ environments in urban areas. Some studies were performed, e.g. in the United States of America [5, 22] or in Germany [27] on state or county level, respectively.

Before analyzing settlement behavior, regulations of the German outpatient care system have to be considered. In Germany, freedom of establishment takes effect for most liberal professions. This applies to physicians and psychotherapists as well. However, to gain accreditation for treating patients insured by statutory health insurances, health care providers have to follow governmental and self- administrational regulations [28, 29]. The insurance together with the 17 associations of statutory health insurance physicians (SHIP) make sure these regulations are adhered to. In order to distribute the health care workforce equally and thus reducing health care costs, physician’s and psychotherapist’s are prohibited to settle down in oversupplied areas. However, metropolises count as singular areas and therefore no inner-city distribution regulation is in place. In this setting the majority of German metropolises are classified as “oversupplied” for most of the physician specialties. An oversupplied area is defined by supplying more than 110 % of demanded physicians/psychotherapists according to national health care plan. The demand is determined individually for each specialty.

Common decision factors for choosing a practice location naturally reflect needs of the individual physician/psychotherapist, e.g. to possess a sufficient client/patient base. On this basis, results of the socioeconomic and demographic evaluation of outpatient care practices’ environments should not substantially be adulterated by governmental and self-administrational regulations.

Aim of the presented Geo-social Analysis of Physician settlement (GAP-Study) is the determination of physicians’ and psychotherapists’ settlement behavior in German metropolises. The focus will be put on socioeconomic and demographic aspects of their practices’ environments.

## Methods

### Study subjects

Physicians and psychotherapists are grouped by specialties - means of various socioeconomic and demographic indicators of their practices’ environments are calculated and compared to each other. For referencing purposes pharmacists’ locations will be chosen. Pharmacists as a non-medical profession within the health care system are similar regulated as physicians and psychotherapists and therefore prone to be used for reference purposes. Hospitals are only marginally involved in the outpatient care in Germany. A minority of hospital physicians is authorized by the SHIP to participate with exactly specified services in the outpatient care. We include hospitals in the GAP-Study as a possible confounding factor for the Geo-social disparities in physician’s settlement. More precisely, whether the distance to the next hospital influences the differing environmental socioeconomics of the various specialties.

**Fig. 1:**
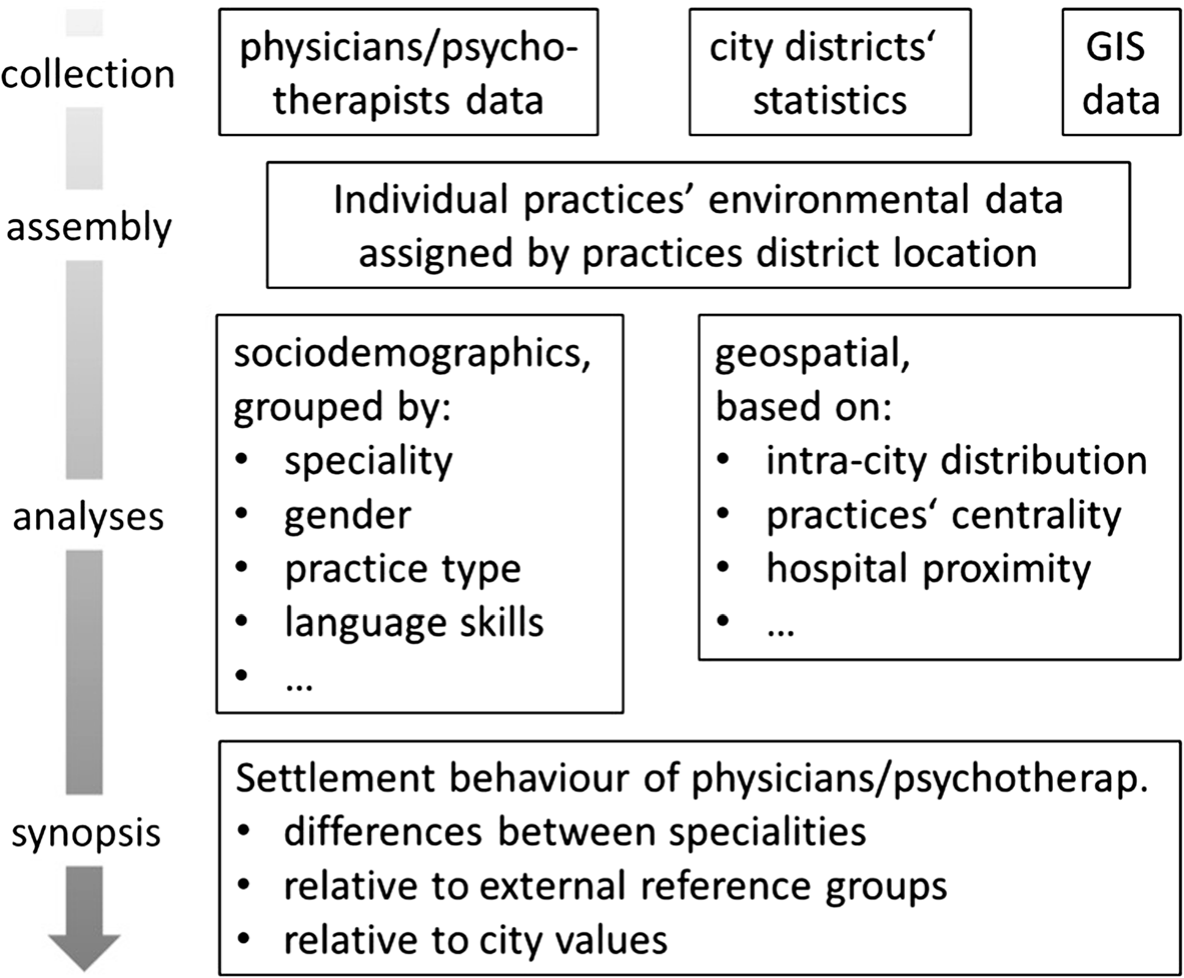
Flow chart of the analyses steps

Beside these statistical analyses physicians’ inner-city distribution will be displayed and analyzed by topographical maps. A flow chart of the analyses steps is shown in Fig. 1.

### Data of health care providers

#### Data source

Data of physicians and psychotherapists within the outpatient care will be collected via the internet-based search engines of the 17 SHIP for the respective region [30]. Not included in most of the Associations are those physicians and psychotherapists treating exclusively self-pay patients (mainly insured by private insurances).

Every SHIP provides his own internet-based search engine. Therefore search procedures and processing of data will be adapted to each SHIP individually. In general, physicians search result lists will be sequentially transferred to Microsoft Word and Excel and reformatted in several steps, to obtain a unified physicians/psychotherapists list.

Pharmacists’ data as the external reference group are publically available through the 17 Federal Chambers of Pharmacists (analogous to SHIP). Similarly, online search engines can be utilized for the transfer of pharmacies’ data.

Official hospital registers are publically available individually for every region. Only those hospitals providing stationary services will be considered.

#### Data collection

Physicians: Following data will be collected: name, surname, title, street, postal code, city, general practitioner or specialist, medical specialty, additional qualification, special authorization, type of practice, number of practice provider and language skills. For the purpose of gender studies sex of each physician will be assigned by the use of her/his first name. However, we are well aware of the limitations identifying one’s gender by use of names. The practice types provided by SHIP according to their legal status are single practices, group practices and medical supply centers. Additionally, the number of participants within a group practice will be recorded too. Language skills will be collected to compare data with the rates of the corresponding ethnic group in their practice environments. Information about language skills as well as gross income data of accredited physicians and psychotherapists are provided by SHIP. Gross income before taxes and insurances are listed for the various specialists, distinguished by practice type (single or group practice). To enhance comprehensiveness, certain specialties will be summarized to categories (Table 1). Specialty categories are derived from official classifications of SHIP and the German Medical Association (GMA) [30].

**Table 1:** Specialty categories comprising more than one speciality

Category	Speciality
Surgery	general, vascular, hand, heart, neurological surgery, plastic & aesthetic surgery, special trauma surgery
General practitioners	General, practical or internal medicine
Internal medicine	w/o subspecialisation (e.g. cardiology), General practitioners excluded
Clinical-theoretical medicine	Pathology, neuropathology, human genetics, biochemistry, laboratory medicine, microbiology & infections epidemiology, transfusion medicine
Neurology/Psychiatry	Neurology, neurology & psychiatry, psychiatry
Psychotherapy, medical	Psychiatry & psychotherapy, psychotherapeutic medicine, psychosomatic medicine & psychotherapy
Radiology (in a broader sense)	Radiology, diagnostic radiology, radiological diagnostics, radiotherapy, nuclear medicine
Others	physical & rehabilitative medicine; mouth, jawbone, face (MJF) surgeons

Pharmacies*:* Following data are available and will be collected: pharmacies name, owner’s name, street, postal code, city and gender.

Hospital: Following data will be collected: hospital name, street, postal code, and city.

#### Data anonymization

Unique identification numbers (ID) will be assigned to comply with data protection and anonymization. Solely the primary practice will remain within the list, double or triple entries of a single physician (up to 2 branch offices per physician are allowed) will be excluded.

### Data of SES on district level

To describe the demographic and socioeconomic characteristics of the study subjects’ neighborhood, official city districts are defined in this project as practices’ environments/neighborhoods. The districts’ population is described by annual-based published reports on their sociological, demographical, and economical characteristics.

Data (such as migration background) are publically available from the corresponding bureau of urban development and statistics. For each metropolis the smallest statistical areas/districts are depicted, where population data are available on a regular basis. The smaller the chosen districts, the more precise intra-city population structure will be reflected. Data of SES will be assigned to the study subjects’ location according to district boundaries via geographical information system (GIS) methods.

### Geographical information system (GIS) methods

City districts will be assigned to the physicians’ entries utilizing geographical information system (GIS) software using district shapefiles. For geospatial analyses the OpenJump [31] and QGIS [32] software will be used. All data will be generated, purchased or reformatted in/to the ESRI shapefile format, based on the World Geodetic System 1984 (WGS 84, EPSG:4326). Most German cities administrative bureaus provide districts, buildings and roads shapefiles. If district digital maps are not available, the internet-based tracking software GPSies [33] will be used in combination with an official district map of the respective city. Collected city districts’ social indicator values will be imported as attributes from an Excel-generated csv-text file. Integration of those indicator values enables their visualization by graduated colored maps.

Geo coordinates of practices of physicians, psychotherapists, and pharmacies will be generated by use of the internet-based “GPSvisualizer” webpage using batch mode [34]. Longitudes and latitudes are displayed here as a tabular list, which will be imported in Microsoft Excel, and reformatted for generating point shapefiles using OpenJump. Accuracy of the generated geo coordinates are controlled on city maps. Dislocated addresses will be processed in a second (non-batch) mode. Following shapefiles will be generated: one file comprising all physicians’ and psychotherapists’ practice locations, one with all pharmacies’ geo coordinates, one with those of the hospitals. For the assembly of shapefile layers on the geospatial maps the QuantumGIS software will be utilized. Post-editing, e.g. labelling and graph arrangements will be performed with Adobe Photoshop 5.0.

Distances of each district center or practice/pharmacy location to the city center will be calculated. Airline distances are calculated by use of the corresponding geo coordinates. Following equation for distance calculations on a sphere will be applied: Distance (km) = ARCCOS [SIN(Latp) * SIN(Latc) + COS(Latp) * COS(Latc) * COS(Lonc- Lonp)] * 6371 km. Whereby the Lat/Lon terms are expressed as radiants, p represents practice/pharmacy or district center, c the city center, and 6371 km the average earth radius.

The same method will be applied for calculating distance to the nearest hospital. By use of GraphPad Prism the shortest distance between district center and hospital will be identified and depicted for the confounder analysis.

### Statistical analysis

For the statistical analysis GraphPad Prism Version 6 will be used.

#### Specialty based analyses

Mean indicator values, standard deviations (SD), and n number are calculated for each specialty. For comparison reasons, the mean ± SD of all accredited physicians and psychotherapists are calculated too. Indicator values of this Total group are inspected whether their distribution follow the Gaussian bell-shaped curve. In terms of a parametric distribution, statistical analyses of differences between the means of more than two groups are performed using the one-way analysis of variance (ANOVA) test in combination with the Bonferroni post-test. In terms of a non-parametric distribution, the Kruskal-Wallis test in combination with the Dunn’s post-test (multiple comparisons) will be carried out.

#### Gender studies

Significant differences between male and female practitioners will be analyzed. In case of a parametric distribution, the unpaired *t*-test (student’s *t*-test) will be chosen, in case of a non-parametric distribution the Mann–Whitney test will be performed.

#### Further statistical analyses

For practice type analyses, means will be calculated for the different practice types grouped by their participant numbers, e.g. classical 2–3 participants’ group practices vs. medical supply centers with more than 10 participants.

Physicians and psychotherapists possessing specific language skills (e.g. Turkish, Russian) will be surveyed whether in their practice environment the abundance of the corresponding ethnic group is above average. Means of those physicians will be compared with the means of the total group and statistically analyzed by unpaired *t*-test or Mann–Whitney test as appropriate.

#### Normalization, ranking and profiling

To enable inter-city comparisons of observed disparities in the settlement behavior of physicians and psychotherapists following analyses steps are performed.

Normalization: The differences of the specialty groups will be normalized to the according city value or the districts’ average, as appropriate. The city value will be defined as 100 %, the speciality values will be expressed as percentage difference (±) thereof. Indicators where no normalization range is applicable, e.g. indicators expressing balance values, will be excluded.

Ranking and Profiling: Alternatively, the specialties will be ranked by their environmental indicator values. Moreover, indicators will be grouped by different topics, like social status, family/children, senior residents, migration background, and urbanity. Significant indicators of each group will be chosen to generate a rank profile for each specialty’s environment. This should lead to a more comprehensive view on environmental data, for both intra-city and inter-city comparisons.

#### Confounder analyses

Potential confounders on district level are the “centrality”, “number of district residents”, and “hospital neighborhood”. These are known to influence the settlement decision of physicians, psychotherapists, and/or pharmacists. Districts’ confounder values are plotted against the district indicator values of interest. Linear regression analyses are performed by using the least square method to test whether a correlation of both variables exists. If such a correlation is present, the confounder adjusted indicator means of the specialties will be calculated on the basis of the regression line’s slope.

## Results and Discussion

With the GAP-Study we want to investigate the German outpatient health care system for gaps of health care provision. We expect the best distribution pattern for the primary care physicians is about 6 physicians per 10.000 residents. Therefore primary care practices should be found in most of the city districts. Rare specialists like radiotherapist with a planed density value of less than 0.1 physicians per 10.000 residents are located particularly more central. To discriminate between such centrality-based alterations of the practices environmental socioeconomics and effective disparities of the health care service, confounder analysis and geo-spatial analysis will be performed.

These analyses will be performed for several German metropolises in order to have more valid data. For rural areas in German the study concept is suitable only to a limited extent, since population statistics are not available on such small-scale level as in metropolises.

The results might serve as discussion basis for an optimization of the practices distribution of physicians and psychotherapists within the outpatient care. The combined statistical and geo-spatial exploration of health services disparities will presumably lead to the development of practice-oriented course of actions within health policy.

Inequalities of health care services are one cause of health inequalities between subgroups of a population. Therefore, reduction of such disparities in health care could lead to a decrease of the widespread problem of social or ethnic dependent imbalances of disease burdens.

## Conclusion

The results of the GAP-Study will answer the following questions:

1. Are disparities of health care providers’ distribution observable in German metropolises regarding the socioeconomic characteristics of their environmental population?
2. Are the primary care physicians whose security of supply is most critical more evenly distributed than the medical specialists?
3. Are specialists such as gynecologists or pediatrics located nearby their clientele, e.g. here women and children?
4. Health inequalities are shown for several diseases. Are the according specialists are found in higher number in areas with residents at higher health risk
5. Does the mean gross income of the medical specialists correlate with that of the neighborhood residents

With these results conclusions can be drawn to if the current distribution of physicians’ in metropolises should be subject for alteration.

## Abbreviations

ANOVA: analysis of variance
GIS: geographical information system
GMA: general medical association
ID: identification numbers
MJF: mouth, jawbone, face
SD: standard deviations
SES: socioeconomic status
SHIP: associations of statutory health insurance physicians

## References

[1] Dussault G, Franceschini MC (2006). Not enough there, too many here: understanding geographical imbalances in the distribution of the health workforce. Hum Resour Heal.

[2] Burton L, Lichter D, Baker R (2013). Inequality, family processes, and health in the “New” rural America. Am Behav Sci.

[3] Guagliardo M, Ronzio C, Cheung I, Chacko E, Joseph J (2004). Physician accessibility: an urban case study of pediatric providers. Heal Place.

[4] Hernandez-Aguado I, Cesteros M, Esteban P (2012). Social inequalities in health and primary care. SESPAS Report 2012. Gac Sanit.

[5] Wan N, Zhan FB, Lu Y, Tiefenbacher JP (2012). Access to healthcare and disparities in colorectal cancer survival in Texas. Heal Place.

[6] World Health Organization (2014). Review of Social Determinants and the Health Divide in the WHO European Region: Final Report.

[7] White K, Borrell LN (2011). Racial/ethnic residential segregation: Framing the context of health risk and health disparities. Heal Place.

[8] Schuster MA, Elliott MN, Kanouse DE, Wallander JL, Tortolero SR, Ratner JA (2012). Racial and ethnic health disparities among fifth-graders in three cities. N Engl J Med.

[9] Lee TC, Glynn RJ, Peña JM, Paynter NP, Conen D, Ridker PM (2011). Socioeconomic status and incident type 2 diabetes mellitus: Data from the Women’s health study. PLoS One.

[10] Chen AY, Escarce JJ (2004). Quantifying income-related inequality in healthcare delivery in the United States. Med Care.

[11] McCarthy M (2013). Two CDC reports look at regional, racial, and sex disparities in US health. BMJ.

[12] Lampert T, Kroll LE, von der Lippe E, Müters S, Stolzenberg H (2013). Socioeconomic status and health: results of the German Health Interview and Examination Survey for Adults (DEGS1). Bundesgesundheitsblatt Gesundheitsforsch Gesundheitsschutz.

[13] Bleich SN, Jarlenski MP, Bell CN, LaVeist TA (2012). Health inequalities: trends, progress, and policy. Annu Rev Public Heal.

[14] Nolasco A, Melchor I, Pina JA, Pereyra-Zamora P, Moncho J, Tamayo N (2009). Preventable avoidable mortality: Evolution of socioeconomic inequalities in urban areas in Spain, 1996–2003. Heal Place.

[15] Michimi A, Ellis-Griffith G, Nagy C, Peterson T (2013). Coronary heart disease prevalence and occupational structure in U.S. metropolitan areas: a multilevel analysis. Heal Place.

[16] Scholes S, Bajekal M, Love H, Hawkins N, Raine R, O’Flaherty M (2012). Persistent socioeconomic inequalities in cardiovascular risk factors in England over 1994–2008: A time-trend analysis of repeated cross-sectional data. BMC Public Health.

[17] Gary-Webb T, Suglia S, Tehranifar P (2013). Social epidemiology of diabetes and associated conditions. Curr Diab Rep.

[18] Vogtmann E, Shanmugam C, Katkoori VR, Waterbor J, Manne U (2013). Socioeconomic status, p53 abnormalities, and colorectal cancer. J Gastrointest Oncol.

[19] Williams DR, Kontos EZ, Viswanath K, Haas JS, Lathan CS, MacConaill LE (2012). Integrating multiple social statuses in health disparities research: The case of lung cancer. Heal Serv Res.

[20] Pampel FC, Krueger P, Denney J (2010). Socioeconomic disparities in health behaviors. Annu Rev Sociol.

[21] Inoue K, Matsumoto M, Toyokawa S, Kobayashi Y (2009). Transition of physician distribution (1980–2002) in Japan and factors predicting future rural practice. Rural Remote Heal.

[22] Mistretta MJ (2007). Differantial effects of economic factors on specialist and family physician distribution in Illions: A country-level analysis. J Rural Heal.

[23] Laditka JN, Laditka SB, Probst JC (2009). Health care access in rural areas: evidence that hospitalization for ambulatory care-sensitive conditions in the United States may increase with the level of rurality. Heal Place.

[24] Larson SL, Fleishman JA (2003). Analyses of national data using urban influence codes: Rural–urban differences in usual source of care and ambulatory service use. Med Care.

[25] Doescher MP, Andrilla CH, Skillman SM, Morgan P, Kaplan L (2014). The contribution of physicians, physician assistants, and nurse practitioners toward rural primary care: Findings from a 13-state survey. Med Care.

[26] Fang H, Chen J, Rizzo JA (2009). Explaining urban–rural health disparities in China. Med Care.

[27] Kistemann T, Schröer M-A (2007). [Small-scale care by SHI physicians and their subjective choice of location within an oversupplied planning area]. Gesundheitswesen.

[28] Widmaier C, Luettge A (2012). Responsibility for healthcare. The national association of the statutory health insurance and long-term care insurance funds.

[29] Statutory health insurance [https://www.gkv-spitzenverband.de/english/statutory_health_insurance/statutory_health_insurance.jsp]

[30] Search engine for phyisicans in Germany [http://www.kbv.de/html/2040.php]

[31] The JUMP Pilot Project (2011). OpenJUMP GIS - the free, java-based open source GIS.

[32] Cavallini P, Piana C (2015). QuantumGIS.

[33] Bechtold K (2014). GPSies - Tracks for Vagabonds.

[34] GPS Visualizer [http://www.gpsvisualizer.com/]

